# Polymorphic Rearrangements of Human Chromosome 9 and Male Infertility: New Evidence and Impact on Spermatogenesis

**DOI:** 10.3390/biom13050729

**Published:** 2023-04-23

**Authors:** Filomena Mottola, Marianna Santonastaso, Valentina Ronga, Renata Finelli, Lucia Rocco

**Affiliations:** 1 Department of Environmental, Biological and Pharmaceutical Sciences and Technologies, University of Campania “Luigi Vanvitelli”, 81100 Caserta, Italy; 2 Department of Woman, Child and General and Special Surgery, University of Campania “Luigi Vanvitelli”, 80138 Napoli, Italy; 3 Prenatal Diagnosis Unit, Varelli Diagnostic Institute, 80126 Napoli, Italy; 4 Create Fertility, London EC2V 6ET, UK

**Keywords:** chromosome 9 polymorphism, chromosome inversion, heterochromatin, sperm aneuploidy, sperm DNA fragmentation, sperm quality, oligozoospermia, genetic infertility, male infertility

## Abstract

Chromosomal polymorphisms are structural variations in chromosomes that define the genomic variance of a species. These alterations are recurrent in the general population, and some of them appear to be more recurrent in the infertile population. Human chromosome 9 is highly heteromorphic, and how its rearrangement affects male fertility remains to be fully investigated. In this study, we aimed to investigate the association between the polymorphic rearrangements of chromosome 9 and male infertility via an Italian cohort of male infertile patients. Cytogenetic analysis was carried out, along with Y microdeletion screening, semen analysis, fluorescence in situ hybridization, and TUNEL assays using spermatic cells. Chromosome 9 rearrangements were observed in six patients: three of them showed a pericentric inversion, while the others showed a polymorphic heterochromatin variant 9qh. Of these, four patients exhibited oligozoospermia associated with teratozoospermia, along with a percentage of aneuploidy in the sperm of above 9%, in particular, an increase in XY disomy. Additionally, high values for sperm DNA fragmentation (≥30%) were observed in two patients. None of them had microdeletions to the AZF loci on chromosome Y. Our results suggest that polymorphic rearrangements of chromosome 9 might be associated with abnormalities in sperm quality due to incorrect spermatogenesis regulation.

## 1. Introduction

Infertility, defined as an incapability of conceiving after one year of unprotected sexual intercourse, is due to male factors in more than 50% of cases [1]. Among the well-known causes of male infertility (lifestyle, environmental factors, acquired disorders, and urogenital tract infections) [2,3], genetic defects are estimated to be responsible for up to 30% of infertility cases [4]. 

Pathological conditions due to genetic defects may result from the altered functionality of one or more genes caused by numerical or structural alterations to chromosomes, as well as mutations in the nucleotide sequence of DNA [5]. The most common type of chromosomal abnormality observed in infertile male patients are Y chromosome long arm microdeletions, as well as aneuploidy of sex chromosomes, which is responsible for Klinefelter syndrome (karyotype 47, XXY) [6]. Chromosomal abnormalities are reportedly more frequently observed in the population of azoo- and/or oligozoospermic males than in the general population [7]. 

Polymorphic variants on chromosomes usually occur in the pericentromeric heterochromatin on the long arms of chromosomes 1, 9, and 16 and the distal heterochromatin of the Y chromosome. Cytogenetic aberrations on chromosome 9 (aneuploidy, deletions, translocations, and inversions) have been reported to be some of the most frequent abnormalities, as its pericentromeric heterochromatin is highly polymorphic in structure, with many intrachromosomal and interchromosomal duplications, containing the largest autosomal amount of heterochromatin [8]. Large blocks of duplications have been identified in the pericentromeric regions at 9p11–12 and 9q11–12/13; nevertheless, 138 gene features have been annotated within it, most of which are pseudogenes, and 16 structures have been identified to include many testicle-specific transcripts [9].

The chromosome 9 centromeric region may have polymorphisms with higher (qh+) or lower (qh−) amounts of heterochromatin or inversions (Inv(9)), with breakpoints preferentially located in the 9p12 and the 9q13–21.1 regions. Particularly, the pericentric inversion of chromosome 9 is one of the most common balanced structural chromosomal aberrations that is found in 1 to 3% of the general population [9,10,11,12]. Although classified as a small chromosomal rearrangement, few studies have highlighted any possible association between genetic alterations to chromosome 9 and recurrent miscarriage [13,14], infertility [15,16,17,18], and reproductive failure [19]. However, genetic studies that investigate the association between chromosomal rearrangements and complex conditions are particularly challenging due to the necessity of analyzing large and heterogeneous cohorts. This explains why the data on this topic are still inconclusive and sometimes contradictory [20]. In this study, we aimed to investigate the presence of polymorphic rearrangements to chromosome 9 and the association of this with infertility in an Italian cohort of infertile male patients. 

## 2. Materials and Methods

### 2.1. Study Design

This study included 96 Italian men (30–41 years old) from the Campania region who were referred to the Reproduction Biology Laboratory (University of Campania “Luigi Vanvitelli”) between April 2022 and September 2022 for infertility due to the inability to conceive after one year of unprotected intercourse. The subjects were all candidates for assisted reproductive technology (ART), nonsmokers, and nondrinkers, and none of them used drugs or underwent any pharmacological therapy. Peripheral blood and seminal fluid were collected for conventional and molecular cytogenetic analysis. In association with the analysis of the karyotype and the evaluation of the basic seminal parameters, fluorescent in situ hybridization (FISH) was performed on the sperm cells for the detection of chromosomal aneuploidies, and the terminal deoxynucleotidyl transferase dUTP nick end labeling (TUNEL) technique was used for the analysis of sperm DNA fragmentation (SDF). Furthermore, Multiplex polymerase chain reaction (PCR) was performed to detect the Y chromosome microdeletions associated with azoospermia and oligospermia. To exclude temporary alterations, analyses of semen parameters, SDF, and sperm FISH were conducted on at least two consecutive samples 3 months apart from each other, collected after sexual abstinence of 3 days (minimum). Female partners were also subjected to cytogenetic testing, with investigations into cystic fibrosis gene mutations and methylenetetrahydrofolate reductase (MTHFR) polymorphisms to exclude female genetic factors from infertility.

### 2.2. Analysis of Karyotype

Cytogenetic analyses were performed on peripheral blood lymphocyte cultures. After collection, blood samples were left to settle for about 3 h. Two mL of whole blood were mixed with 8 mL of complete medium (Lymphochrome, Lonza Bioscience, Morrisville, NC, USA). After 72 h of incubation at 37 °C, a 1.5 µg/mL colcemid solution (N-desacetyl-N-methylocolchicine, Gibco, Thermo Fisher Scientific, Waltham, MA, USA) was added to 10 mL of blood solution and culture medium for 45 min. Then, the solution was centrifuged (2500 rpm, 5 min), the pellet was resuspended with a hypotonic solution (KCl, 0.075 M, VWR Chemicals, Radnor, PA, USA), centrifuged again, and finally, a 3:1 methanol/glacial acetic acid (VWR Chemicals, Radnor, PA, USA) fixation was performed. A concentrated cell suspension was loaded onto the slides and dried on a slide warmer at 70 °C overnight. Chromosomes were analyzed by G-Trypsin-Giemsa (GTG) banding technique [21]. Briefly, the slides were placed in a solution of 2.5% trypsin (Microgem, Naples, Italy) and 0.9% Na-saline (APPLICHEM GmbH, Darmstadt, Germany) solution for 3 min. The slides were then immersed for 10 s in a phosphate solution (Gurr’s buffer solution pH 6.8, Thermo Fisher Scientific, Rodano, MI, Italy) and stained in a 6% Giemsa (Sigma Aldrich, St. Louis, MI, USA) solution for 5 min. The slides were air-dried and examined under a light microscope (Nikon Eclipse E-600). Metaphases were karyotyped and interpreted according to the International System for Human Cytogenetic Nomenclature [22].

The criteria used to evaluate the heterochromatic variants qh+, qh++, and qh− of chromosome 9 were that the variant must be at least 2–3 times or half the size of the corresponding region on its homolog in all metaphases examined [23]. The analysis was carried out by the fully automated cytogenetic karyotyping software Genikon ver.3.7, from acquisition to karyotyping. At least 50 metaphases were analyzed for each patient. 

### 2.3. Y Microdeletion Detection

The screening for Y chromosome microdeletions was performed using a Y microdeletion Kit (Nuclear Laser Medicine, Settala, MI, USA), according to Simoni et al. [24]. Briefly, genomic DNA was extracted from EDTA-peripheral blood samples using the DNA Extraction Kit (Qiagen, Hilden, Hilden, Germany) and amplified by a Multiplex PCR. The PCR protocol consisted of an initial denaturing step of 4 min at 95 °C, followed by 25 cycles at 94 °C for 30 s, 57 °C for 30 s, and 70 °C for 1 min. Cycling was concluded with a final extension at 72 °C for 7 min. For each PCR assay, we incorporated the following samples as controls: genomic DNA from a normal fertile man, genomic DNA from a normal fertile woman, and a PCR mixture without DNA (blank control). To confirm amplification, all samples were amplified twice. As primers, we used three oligonucleotides that amplify the sequence-tagged site regions for AZFa (sY84 and sY86), AZFb (sY127 and sY134), and AZFc (sY254 and sY255) on the Y chromosome.

### 2.4. Semen Analysis

Semen analysis was conducted according to the World Health Organization (WHO) guidelines [25]. Semen samples were collected in a sterile container and kept at 37 °C until analysis. After fluidification, sperm concentration and progressive and nonprogressive sperm motility were assessed by counting about 200 spermatozoa for each patient using a Makler counting chamber (Securlab, ROME), while sperm morphology was evaluated by using prestained slides (Test-simplets^®^ stained slides (Origio; Cooper Surgical, Inc., Måløv, Denmark) [26]. 

### 2.5. Fluorescence In Situ Hybridization Analysis

FISH was performed by using a commercial kit (AneuVysion Multicolor DNA probe kit, Abbott, Abbott Park, IL, USA) to identify specific aneuploidy for chromosomes X, Y, 13, 18, 21. Semen samples were diluted in phosphate-buffered saline (PBS, Lonza Bioscience, Morrisville, NC, USA) and washed three times by centrifugation for 5 min at 2000 rpm. The pellet was resuspended in fresh fixative (3:1; methanol:acetic acid). The suspension was spread onto clean glass slides and air-dried. The sperm nuclei were decondensed and denatured by incubation in 1 M NaOH (APPLICHEM GmbH, Darmstadt, Germany) for 5 min at room temperature, dehydrated in ethanol series (70%, 95%, and 100%, 2 min each), and air-dried. The probe mixture (Centromeric—CEP 18/X/Y; Locus specific—LSI 13/21) was denatured at 72 °C for 10 min. After application of the probe mixture onto the slide, hybridization was performed overnight at 37 °C in a moist chamber. The slides were washed for 2 min at 72 °C in 0.4% and 2% saline sodium citrate (APPLICHEM GmbH, Darmstadt, Germany) and then stained with 4′,6-diamidino-2-phenyl-indole (DAPI) containing antifade medium 1,4-diazabicyclo[2.2.2]octane (DABCO) (Sigma Aldrich, St. Louis, MI, USA). The slides were examined with an epifluorescence microscope (Nikon Eclipse E-600, Melville, New York, USA) equipped with filter sets optimized for DAPI, FITC, Texas Red, and Aqua. Cells were captured with a CCD camera using Genikon Imaging System (Nikon Instruments). Same-color signals were counted as two if they were separated by at least one signal diameter and had the same intensity, size, and shape.

### 2.6. Terminal Deoxynucleotidyl Transferase dUTP Nick End Labeling Assay (TUNEL)

TUNEL assay was conducted according to Iovine et al. [27] by using the In Situ Cell Death Detection Kit (Roche Diagnostics). Spermatozoa were washed in 1× PBS. The sperm suspension was fixed with 4% paraformaldehyde (Sigma Aldrich, St. Louis, MI, USA) for 1 h at room temperature. Cells were washed again in 1× PBS and permeabilized with 0.5% Triton X-100 in 0.1% sodium citrate (Sigma Aldrich, St. Louis, MI, USA) for 1 h on ice. The permeabilized spermatozoa were washed once in 1× PBS and incubated with the TUNEL reaction mixture, containing terminal deoxynucleotidyl transferase (TdT) plus labeled dUTP, in a moist chamber at 37 °C for 1 h in the dark. After labeling, the cells were washed in 1× PBS and counterstained by DAPI to visualize the undamaged nuclei. Negative (no TdT enzyme in the reaction mixture) and positive (cells previously treated with DNAse I, 1 mg/mL for 30 min at room temperature) controls were included in the analysis. Sperm cells were analyzed with an epifluorescence microscope (Nikon Eclipse E-600) equipped with filter sets optimized for DAPI and FITC. Cells were captured with a CCD camera using the Genikon Imaging System. The final percentage of sperm with fragmented DNA was defined as percentage of sperm DNA fragmentation (% SDF).

### 2.7. Statistical Analysis

Data among the experimental groups were compared using ANOVA testing via GraphPad Prism 6 (San Diego, CA, USA) and are expressed as mean ± standard deviation (SD). The results were considered statistically significant for *p* ≤ 0.05. All experiments were performed in triplicate.

## 3. Results

### 3.1. Karyotype Analyses

**Figure 1:**
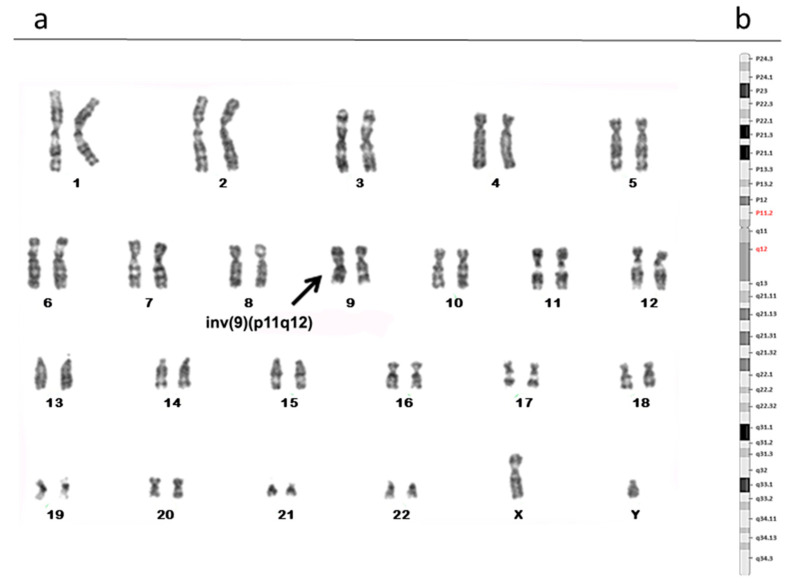
Karyotype analysis showing a pericentric inversion of chromosome 9: 46, XY, inv(9) (p11q12) (**a**). Karyogram including the bands of chromosome 9; in red those involved in the rearrangement (**b**).

**Figure 2:**
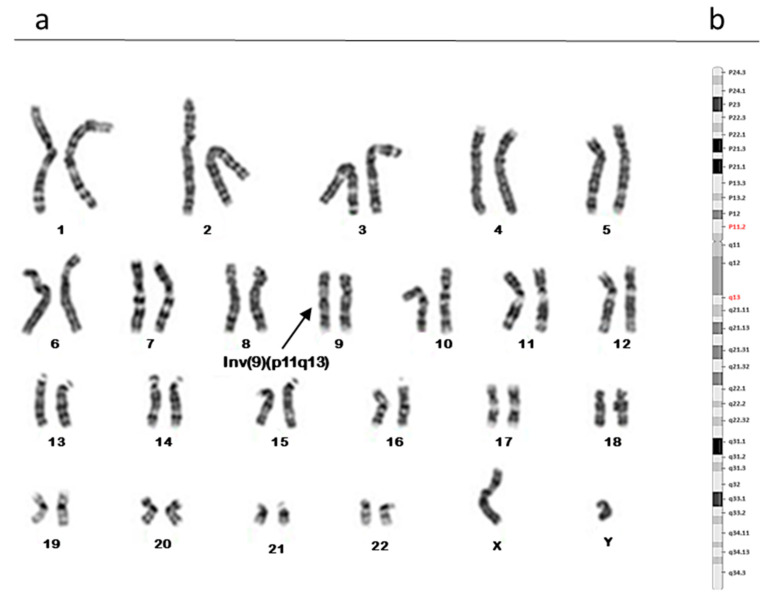
Karyotype analysis showing a pericentric inversion of chromosome 9: 46, XY, inv(9) (p11q13) in a heterozygous condition (**a**). Karyogram including the bands of chromosome 9; in red those involved in the rearrangement (**b**).

**Figure 3:**
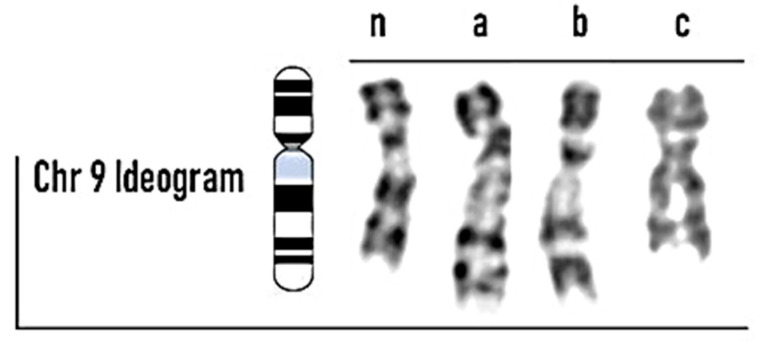
Polymorphic chromosome 9 variant 9qh (n: normal; a: qh++; b: qh+; c: qh−).

The karyotype analyses identified 6 out of 96 patients (6.25%) with polymorphic rearrangements to chromosome 9. We detected three cases that showed a pericentric inversion of chromosome 9: specifically, it was 46, XY, inv(9) (p11q12) in two patients (Figure 1), while one case showed the breakpoint in p11q13 (Figure 2). The other three patients showed a polymorphic variant 9qh (qh++, qh+, and qh−) (Figure 3 (a; b; c)) in a heterozygous condition. None of the female partners of the patients under examination presented chromosomal alterations.

### 3.2. Y Microdeletion Screening

**Figure 4:**
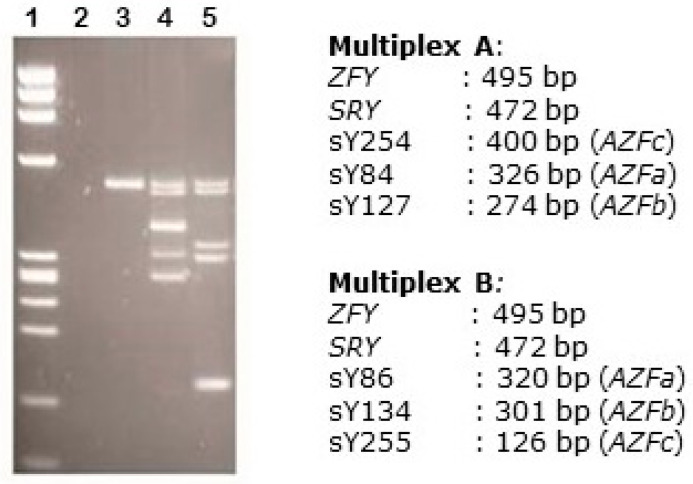
Multiplex PCR products obtained using a mixture of primers. Lane 1: Phi-X174 RF DNA Hae III Digest molecular weight marker; lane 2: water; lane 3: control female DNA; lanes 4 and 5: DNA of a patient examined amplified with multiplexes A and B, respectively. The presence of all investigated bands indicates the absence of microdeletions.

There was no patient with microdeletions to the AZF loci on chromosome Y (Figure 4).

### 3.3. Standard and Molecular Analysis of Seminal Ejaculates

**Figure 5:**
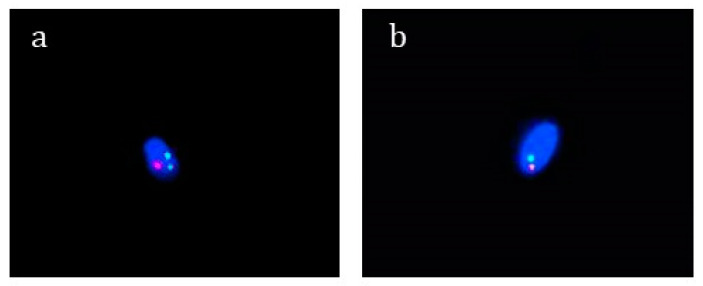
Representative image of FISH signals on aneuploid spermatozoa of patients carrying chromosome 9 rearrangements, analyzed by fluorescence microscope. CEP 18/X/Y probes were used to detect alpha satellite sequences in the centromeric regions of chromosomes 18, X and Y. Chromosome X appears in green, chromosome Y in red, and chromosome 18 in sky-blue (**a**). LSI 13/21 probes were instead utilized to detect the 13q14 region and the 21q22.13 to 21q22.2 regions. Chromosome 13 appears in green, while chromosome 21 appears in red (**b**).

**Figure 6:**
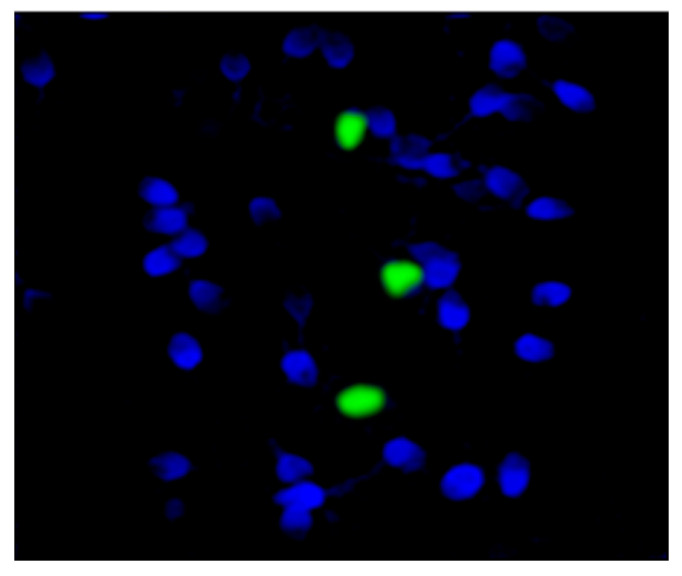
Representative image of sperm cells with fragmented DNA (green fluorescence) and sperm cells with intact DNA (blue fluorescence), analyzed by fluorescence microscope.

Four out of six patients with chromosome 9 rearrangements (with pericentric inversion and polymorphic variant 9qh++) showed remarkable alterations to semen quality; in particular, moderate/severe oligospermia associated with teratozoospermia (<4% of morphologically normal sperm) was observed (Table 1). The FISH analysis highlighted a percentage of aneuploidy in the sperm cells from 9.0% to 11.0% in 4 out of six patients with chromosome 9 rearrangements (pericentric inversion and polymorphic variant 9qh++), in particular, an increase in sex chromosomes disomy (Table 1 and Figure 5). Two patients with a pericentric inversion of chromosome 9 also showed high levels of sperm DNA fragmentation (≥30%) [28] (Table 1 and Figure 6).

## 4. Discussion

In this study, we investigated the association between polymorphic rearrangements of chromosome 9 with male infertility, identifying patients (6.25% of our investigated cohort) with chromosome 9 rearrangements (pericentric inversion and polymorphic variants) and remarkable alterations to semen quality (in 4.17% of carriers). The incidence of these abnormalities in infertile patients was higher than the reported value of 1–3% in the normal population, suggesting that they could affect male fertility. According to our results, in these individuals, the structural rearrangements seem to influence the sperm count and morphology, as well as meiosis and DNA integrity, with the percentage of total sperm aneuploidies between 9 and 12% and the values for sperm DNA fragmentation above 30%. Chromosomal structural rearrangements have been previously associated with a higher rate of sperm DNA fragmentation [29], which is a well-known cause of male infertility [30]. High levels of sperm DNA fragmentation have also been related to teratozoospermia [31] and reduced fertility, with an increased incidence of miscarriage and reduced live birth rate after ICSI [32].

Half of the patients showed pericentric inversions of chromosome 9. Pericentric inversions are intrachromosomal structural rearrangements whereby two breaks occur on both sides of the centromere, allowing the chromatin fragment to rotate by 180 degrees [33]. Most pericentric inversion carriers have a normal phenotype and usually normal fertility. However, some of them can have difficulties in conceiving normal offspring because of the production of chromosomally unbalanced gametes following abnormal meiotic events [16]. Hence, when the pairing of an inverted chromosome with its normal homolog implies the formation of an inversion loop, the occurrence of a different number of genetic recombinations within the loop leads to the formation of two abnormal chromosomes that are duplicated and deleted at the regions outside of the inversion. According to the size of the unbalanced chromosomal segment, such recombinant chromosomes may lead to either spontaneous abortions or abnormal children [34,35].

The research in this area has become more clinically relevant in the past few years with the advent of intracytoplasmic sperm injection (ICSI). ICSI has been extremely successful in the treatment of male infertility, but the transmission of cytogenetic defects to the offspring is a major concern [36]. A recent study conducted in China investigated the clinical outcomes of 107 couples with one inversion of chromosome 9 in at least one partner undergoing IVF or ICSI (compared to couples with normal karyotype) [37]. Regardless of the type of technique chosen (IVF or ICSI), the authors observed no difference between the groups when fertilization, implantation, clinical pregnancy, miscarriage, and live birth rates were examined. Moreover, pre-implantation genetic testing was used to analyze the embryos created by couples with pericentric chromosome 9 variants. The study revealed that unbalanced structural rearrangements were not inherited by the embryos, excluding an association between chromosome 9 inversion and an increased risk of unbalanced chromosomes [20]. Hence, although they appear to be related to male infertility, assisted reproductive outcomes do not seem to be affected by chromosome 9 rearrangements. Sperm parameter alterations in chromosome 9 rearrangement carriers are, in part, bypassed by assisted reproductive techniques, thanks to the artificial selection of the best sperm cell, and this would explain why chromosome 9 polymorphisms do not affect assisted reproductive outcomes.

Polymorphisms of chromosome 9 are known to have no effect on the phenotype because they mainly involve constitutionally inactive heterochromatic regions. The precise role of heterochromatin in the human genome remains undefined; however, the dysregulation of heterochromatin is associated with severe disease phenotypes due to epigenetic defects of particular genes [38]. Altered heterochromatin contents have been associated with genetic syndromes, recurrent miscarriages, and infertility in men [23]. Although the clinical significance of the heteromorphisms is still not well understood, it was already demonstrated several years ago that constitutive heterochromatin is important for gene regulation [39].

SPATA31A5 and its paralogue SPATA31A7 are spermatogenesis-associated gene family members located in the q12 region of chromosome 9 [40]. SPATA genes are involved in cell differentiation and sex determination [41] and play an important role in male fertility. They are mainly expressed in the testis and intervene in the fundamental functions of spermatogenesis; in fact, their correct expression guarantees the production of morphologically mature and motile spermatozoa [42,43]. In 2015, Wu et al. found that sperm concentration was significantly reduced in SPATA31 knock-out mice because of premature germ cell shedding into the seminiferous tubule and epididymis lumen [44]. Significant differences in the methylation status of SPATA genes were found between oligozoospermic and normozoospermic individuals; in particular, hypermethylation appears to be the cause of decreased sperm count [45]. Although the functions and expression of the SPATA31 subfamily A member 5 and member 7 are not well studied, these could play a role in spermatogenesis as well [46].

In agreement with studies involving candidate couples for assisted reproduction, our data suggest that chromosome 9 polymorphisms, including variations in the length of the heterochromatic region, could be associated with semen abnormalities due to the altered expression of SPATA genes, resulting in defective meiotic segregation. This hypothesis is supported by the evidence that the altered formation of heterochromatin leads to the aberrant activation of genes located immediately close by [38].

Therefore, the meiotic alteration would lead to an increase in sperm aneuploidies as well as a reduction in sperm concentration due to the natural apoptotic selection of genetically altered sperm cells. Furthermore, it has been demonstrated that, in carriers of a balanced structural abnormality, sperm DNA fragmentation is closely related to the presence of the aberration itself, probably due to impaired chromatin reorganization containing unrepaired breaks. Hence, a meiotic checkpoint would eliminate some defective germ cells for apoptosis while others would escape this mechanism, resulting in the presence of a few genetically altered cells in the ejaculate [47].

Moreover, the absence of Y chromosome microdeletions excludes a possible contributing cause to infertility in the same patients. Major concerns regarding the involvement of chromosomal rearrangements in male infertility have been identified in chromosome 9 pericentromeric inversions and 9qh++ heteromorphism. This could depend on the fact that the 9qh− and 9qh+ heteromorphisms, being less extensive, do not cover the 9q12 region, which is critical for the presence of the SPATA genes. Certainly, further studies will be needed to clarify the effective role of SPATA genes in the onset of the infertile phenotype in carriers of chromosome 9 polymorphisms, such as the methylation and sequence analysis of these genes, FISH experiments to establish if and how these particular genes are involved in chromosomal rearrangements, and their association with chromosomal rearrangements, and these represent new perspectives for the identification of a genetic cause of male infertility.

In conclusion, our results suggest that the polymorphic variants of chromosome 9 may often cause infertility or sub-fertility in men due to spermatogenic disturbances, highlighting the importance of appropriate genetic counseling in case of infertility. The accurate diagnosis of the constitutional karyotype is here confirmed as a valuable aid in the early management of patients who undertake fertility evaluation, and, as already suggested by Madon et al. in 2005 [23], chromosome polymorphic variants should not be ignored by cytogeneticists and clinicians as heterochromatin may play an important cellular role that is not yet known.

## Figures and Tables

**Table 1:** Sperm parameters, percentage of spermatic aneuploidy, and sperm DNA fragmentation (SDF) in six patients carrying polymorphic variations of chromosome 9.

Age	Karyotype	Sperm Concentration /mL	Normal Sperm Morphology (%)	Spermatic Aneuploidy (%)	SDF (%)
34	46, XY, inv(9) (p11;q12)	10.0 × 10^6^	3	9.7	19
41	46, XY, inv(9) (p11;q13)	0.8 × 10^6^	1	11.0	38
36	46, XY, 9qh+	32.0 × 10^6^	6	3.1	19
33	46, XY, 9qh++	14.0 × 10^6^	2	9.0	12
38	46, XY, 9qh−	36.0 × 10^6^	10	3.0	16
30	46, XY, inv(9) (p11;q12)	11.0 × 10^6^	1	10.4	40

## Data Availability

Not applicable.
